# 老年套细胞淋巴瘤的临床特征和预后影响因素

**DOI:** 10.3760/cma.j.issn.0253-2727.2023.06.009

**Published:** 2023-06

**Authors:** 晓宇 郝, 萍 杨, 薇 张, 辉 刘, 秀华 孙, 秀斌 肖, 景文 王, 振玲 李, 利红 李, 树叶 王, 娟 何, 晓玲 李, 红梅 景

**Affiliations:** 1 北京大学第三医院，北京 100191 Peking University Third Hospital, Beijing 100191, China; 2 北京协和医院，北京 100730 Peking Union Medical College Hospital, Beijing 100730, China; 3 北京医院，北京 100730 Beijing Hospital, Beijing 100730, China; 4 大连医科大学附属第二医院，大连 116027 The Second Hospital of Dalian Medical University, Dalian 116027, China; 5 解放军总医院第五医学中心，北京 100039 The 5th Medical Center of PLA General Hospital, Beijing 100039, China; 6 北京同仁医院，北京 100730 Beijing Tongren Hospital, Beijing 100730, China; 7 中日友好医院，北京 100029 China-Japan Friendship Hospital, Beijing 100029, China; 8 北京清华长庚医院，北京 102218 Beijing Tsinghua Changgung Hospital, Beijing 102218, China; 9 哈尔滨医科大学附属第一医院，哈尔滨 150001 The First Hospital of Harbin Medical University, Harbin 150001, China; 10 中国医科大学附属第一医院，沈阳 110001 The First Hospital of China Medical University, Shenyang 110001, China; 11 辽宁省肿瘤医院，沈阳 110042 Liaoning Cancer Hospital&Institute, Shenyang 110042, China

**Keywords:** 套细胞淋巴瘤, 老年, 临床特点, 预后, Mantle cell lymphoma, Elderly, Clinical features, Prognosis

## Abstract

**目的:**

分析老年（≥65岁）套细胞淋巴瘤（MCL）患者的临床特征和预后影响因素，探讨基础营养和疾病状态对老年MCL患者预后的影响。

**方法:**

回顾性分析2000年1月至2021年2月在北京大学第三医院等11个中心收治的255例老年MCL患者的临床资料，主要指标包括年龄、性别、MIPI评分、治疗方案及疗效，采用Kaplan-Meier法和Cox回归模型进行预后的单因素及多因素分析；对50例有白蛋白水平及基础疾病资料的老年MCL患者进行老年综合疾病评估，分析老年患者基础疾病和基础营养状态，以及老年综合疾病状态对预后的影响。

**结果:**

在纳入的795例MCL患者中，老年MCL患者255例（32.1％）。老年MCL好发于男性（78.4％），中位年龄69（65～88）岁，初诊时多为晚期（88.6％）。老年MCL患者的治疗总有效率为77.3％，完全缓解率为33.3％。3年总生存（OS）率为42.0％，3年无进展生存（PFS）率为21.2％。老年MCL患者相较年轻患者更易合并高血压等慢性基础疾病。多因素分析结果显示，与PFS期相关的独立预后不良因素包括高龄（*P*＝0.021）、Ann Arbor分期Ⅲ～Ⅳ期（*P*＝0.003）、高LDH水平（*P*＝0.003）、骨髓受累（*P*＝0.014）；与OS期相关的独立预后不良因素包括高龄（*P*＝0.001）、高LDH水平（*P*＝0.003）。老年综合疾病评估提示肾功能不全为与OS相关的预后不良因素（*P*＝0.047）。

**结论:**

老年MCL患者合并基础疾病更多；高龄、高LDH水平、肾功能不全、骨髓受累、Ann Arbor分期Ⅲ～Ⅳ期为老年MCL患者预后的独立危险因素。

## Abstract (en)

**Objective:**

To examine the clinical characteristics and prognostic factors of elderly patients with mantle cell lymphoma（MCL）and the impact of nutrition and underlying diseases on the prognosis of elderly patients with MCL.

**Methods:**

retrospectively analyzed 255 elderly patients with MCL from 11 medical centers, including Peking University Third Hospital between January 2000 and February 2021. We analyzed clinical data, such as age, gender, Mantle Cell Lymphoma International Prognostic Index score, and treatment options, and performed univariate and multivariate prognostic analysis. We performed a comprehensive geriatric assessment on elderly MCL patients with medical records that included retraceable underlying disease and albumin levels, and we investigated the impact of basic nutrition and underlying disorders on MCL prognosis in the elderly.

**Results:**

There were 255 senior individuals among the 795 MCL patients. Elderly MCL was more common in males (78.4％), with a median age of 69 yr (ages 65–88), and the majority (88.6％) were identified at a late stage. The 3-yr overall survival (OS) rate was 42.0％, with a 21.2％ progression-free survival (PFS) rate. The overall response rate (ORR) was 77.3％, with a 33.3％ total remission rate. Elderly patients were more likely than younger patients to have persistent underlying illnesses, such as hypertension. Multivariate analysis revealed that variables related with poor PFS included age of ≥80 (*P*＝0.021), Ann Arbor stage Ⅲ–Ⅳ (*P*＝0.003), high LDH level (*P*＝0.003), involvement of bone marrow (*P*＝0.014). Age of ≥80 (*P*＝0.001) and a high LDH level (*P*＝0.003) were risk factors for OS. The complete geriatric assessment revealed that renal deficiency was associated with poorer OS (*P*＝0.047).

**Conclusion:**

Elderly MCL patients had greater comorbidities. Age, LDH, renal function, bone marrow involvement, and Ann Arbor stage are all independent risk factors for MCL in the elderly.

套细胞淋巴瘤（MCL）好发于老年人[1]，老年患者的基础健康状况相对较差，合并心肺基础疾病多，对高剂量化疗方案及自体造血干细胞移植耐受不佳，其治疗仍面临巨大的挑战。目前对中国老年MCL患者临床特点、治疗方案及生存现状进行分析的报道较少。本研究回顾性分析2000年1月至2021年2月在北京大学第三医院、北京协和医院、中日友好医院等11个中心收治的255例老年MCL患者（年龄≥65岁），总结分析其临床特点、治疗方案及预后影响因素；对其中可回顾基础营养状况及基础疾病的50例老年MCL患者进行老年综合疾病状态评估，分析老年MCL患者的营养状态和基础疾病及其对预后的影响。

## 病例与方法

1. 病例：本研究收集2000年1月至2021年2月北京大学第三医院、解放军总医院第五医学中心、北京协和医院、中国医科大学附属第一医院、中日友好医院、北京同仁医院、北京医院、北京清华长庚医院、大连医科大学附属第二医院、哈尔滨医科大学附属第一医院、辽宁省肿瘤医院共11个中心收治的795例MCL患者的临床和随访资料，其中255例为老年MCL患者（年龄≥65岁），所有患者均根据2008年WHO分类标准确诊。治疗前检查包括体格检查、外周血细胞计数及分类、肝肾功能、血清乳酸脱氢酶（LDH）、白蛋白、骨髓象等。治疗前所有病例按照Ann Arbor标准进行分期，按照美国东部肿瘤协作组（ECOG）标准进行体力状态评分，根据MCL国际预后评分系统（MIPI）和结合Ki-67指数的MCL国际预后评分系统（MIPI-c）进行评分。观察指标包括性别、年龄、Ki-67指数、B症状、侵犯器官、LDH、白蛋白等。

在全部795例MCL患者中，共122例MCL患者可回顾性分析基础疾病及基础营养状态，其中老年MCL患者（年龄≥65岁）50例。对这50例老年MCL患者进行老年综合疾病评估，纳入分析的生化指标包括白蛋白、肝功能、肾功能，观察的疾病包括高血压、糖尿病、冠心病、脑血管疾病、慢性乙型肝炎以及合并其他恶性肿瘤。

2. 治疗方案：255例老年MCL患者中，126例初治时接受R-CHOP方案（利妥昔单抗、环磷酰胺、阿霉素、长春新碱、泼尼松）；39例接受非强化方案，包括BR（利妥昔单抗、苯达莫司汀）及VR-CAP（硼替佐米、利妥昔单抗、环磷酰胺、阿霉素、泼尼松）；46例接受强化方案，包括R-CHOP/DHAP（利妥昔单抗、环磷酰胺、阿霉素、长春新碱、泼尼松；利妥昔单抗、顺铂、阿糖胞苷、地塞米松）、R-HyperCVAD（利妥昔单抗、环磷酰胺、长春新碱、阿霉素、地塞米松）、R-EPOCH（利妥昔单抗、阿霉素、依托泊苷、长春新碱、环磷酰胺、泼尼松）及R-high AraC（包含利妥昔单抗、高剂量阿糖胞苷）；39例接受IR方案（伊布替尼、利妥昔单抗）或类似方案（R2方案：利妥昔单抗、来那度胺；IR2方案：伊布替尼、利妥昔单抗、来那度胺）；5例接受其他类型方案治疗。

3. 疗效评价标准：按照淋巴瘤疗效评价标准，分为完全缓解（CR）、部分缓解（PR）、疾病稳定（SD）、疾病进展（PD）。总生存（OS）期为患者首次确诊至死亡或末次随访的时间，无进展生存（PFS）期指从治疗开始至淋巴瘤进展或复发或末次随访的时间。

4. 随访：采用电话随访方式，中位随访时间57.0个月，随访截止日期为2021年2月。

5. 统计学处理：采用SPSS 27.0软件进行统计学处理，计数资料比较采用*χ*^2^检验，以Kaplan-Meier法进行预后的单因素分析，行Log-rank检验；应用Cox回归模型进行预后的多因素分析。以*P*<0.05为差异有统计学意义。

## 结果

1. 老年MCL一般临床特征：在全部的795例MCL患者中，≥65岁的老年患者共255例（32.1％）。老年患者的中位发病年龄为69（65～88）岁，其中男200例，女55例，男女比3.6∶1。基线ECOG评分52.1％的患者为0分，36.7％的患者为1分，11.2％的患者≥2分；40.4％的患者存在B症状。88.6％的老年MCL患者起病时即为晚期（Ⅲ～Ⅳ期），27.8％的患者Ki-67≥50％。40.8％的患者起病时LDH升高。病理分型方面，在老年MCL患者中，217例（85.1％）为经典型，10例为惰性MCL（3.9％），28例（11.0％）为多形性或母细胞型。淋巴瘤结外受累方面，228例（89.4％）老年MCL患者有结外受累，141例（55.3％）有骨髓受累，67例（26.3％）有胃肠道受累，98例（38.4％）有脾脏受累，40例（15.7％）有口咽淋巴环侵犯。根据MIPI进行评分，55例（21.6％）老年MCL患者MIPI评分为低危，114例（44.7％）为中危，86例（33.7％）为高危；应用MIPI-c进行评分，108例（42.4％）患者为低危～低中危，82例（32.2％）患者为中高危，65例（25.5％）患者为高危。

2. 老年基础疾病：在全部795例MCL患者中，122例可回顾性分析起病时基础疾病及基础营养状态，其中≥65岁的老年MCL患者50例。122例患者的基础疾病合并情况和基础营养状态见表1。白蛋白可反映患者的基础营养状况，在该122例MCL患者中，仅8.3％的年轻患者初诊时有低白蛋白血症，而18.0％的老年患者白蛋白低于正常低限。36.1％的年轻MCL患者存在基础疾病，而老年MCL患者中56.0％合并基础疾病，比例显著高于年轻患者（*P*＝0.041）；老年患者中高血压、脑血管病、冠心病患病率及合并其他恶性肿瘤的比例均高于年轻MCL组患者（*P*值均<0.05）。

**表1:** 122例套细胞淋巴瘤（MCL）患者的基础疾病及基础营养状态［例（％）］

指标	年轻（年龄<65岁）MCL（72例）	老年（年龄≥65岁）MCL（50例）	*χ*^2^值	*P*值
白蛋白			2.557	0.160
正常	66（91.7）	41（82.0）		
减低	6（8.3）	9（18.0）		
合并基础疾病			4.731	0.041
否	46（63.9）	22（44.0）		
是	26（36.1）	28（56.0）		
合并≥2种基础疾病			0.511	0.606
否	60（83.3）	44（88.0）		
是	12（16.7）	6（12.0）		
肾功能不全			0.100	0.758
否	66（91.7）	45（90.0）		
是	6（8.3）	5（10.0）		
肝功能不全			4.323	0.046
否	63（87.5）	49（98.0）		
是	9（12.5）	1（2.0）		
糖尿病			0.940	0.522
否	64（88.9）	47（94.0）		
是	8（11.1）	3（6.0）		
高血压			7.086	0.011
否	60（83.3）	31（62.0）		
是	12（16.7）	19（38.0）		
脑血管疾病			1.721	0.226
否	70（97.2）	46（92.0）		
是	2（2.8）	4（8.0）		
乙肝			0.011	1.000
否	68（94.4）	47（94.0）		
是	4（5.6）	3（6.0）		
冠心病			3.281	0.158
否	71（98.6）	46（92.0）		
是	1（1.4）	4（8.0）		
合并其他肿瘤			3.281	0.158
否	71（98.6）	46（92.0）		
是	1（1.4）	4（8.0）		

3. 疗效：255例老年MCL患者的治疗总反应率（ORR，CR率+PR率）为76.9％，33.3％的患者达到CR。170例（66.7％）患者为复发难治，其中复发患者113例（44.3％），难治患者57例（22.4％）。在全部255例老年MCL患者中，近一半（49.4％）以R-CHOP方案作为初始治疗方案，接受R-CHOP方案的老年MCL患者的3年PFS率为23.0％，3年OS率为46.8％；15.3％的患者接受了包含伊布替尼或来那度胺的初治治疗方案（包括IR方案、R2方案、IR2方案），3年PFS率为0，3年OS率为2.6％。共有116例（45.5％）患者进行了维持治疗，维持治疗方案包括利妥昔单抗（51例，20.0％）、来那度胺（12例，4.7％）、伊布替尼（21例，8.2％）及IR/R2（12.5％）；接受维持治疗组3年PFS率高于无维持治疗组（27.6％对15.8％，*P*＝0.022）。

4. 预后影响因素分析：全部255例老年MCL患者中，中位随访时间为57.0个月，平均PFS期为24.3个月，3年PFS率为21.2％，5年PFS率为8.6％；平均OS期为35.7个月，3年OS率为42.0％，5年OS率为14.1％（图1）。对患者进行预后因素分析，纳入因素包括年龄、性别、病理分型、Ann Arbor分期、ECOG评分、B症状、Ki-67指数、LDH、结外受累、骨髓侵犯、消化道侵犯、口咽侵犯、脾脏受累、MIPI及MIPI-c评分等。单因素分析显示，与PFS相关的危险因素包括年龄≥80岁（*P*＝0.015）、Ann Arbor分期为Ⅲ～Ⅳ期（*P*＝0.001）以及高LDH水平（*P*＝0.001）；与OS相关的危险因素包括年龄≥80岁（*P*<0.001）、男性（*P*＝0.005）、Ki-67≥50％（*P*＝0.001）、ECOG评分≥2分（*P*＝0.034）以及高LDH水平（*P*＝0.001）。将以上因素纳入Cox回归模型进行预后的多因素分析，结果显示，高龄（年龄≥80岁）、Ann Arbor分期Ⅲ～Ⅳ期、高LDH水平、骨髓受累为与PFS相关的预后不良因素；年龄≥80岁及高LDH水平为与OS相关的预后不良因素（表2）。

**图1:**
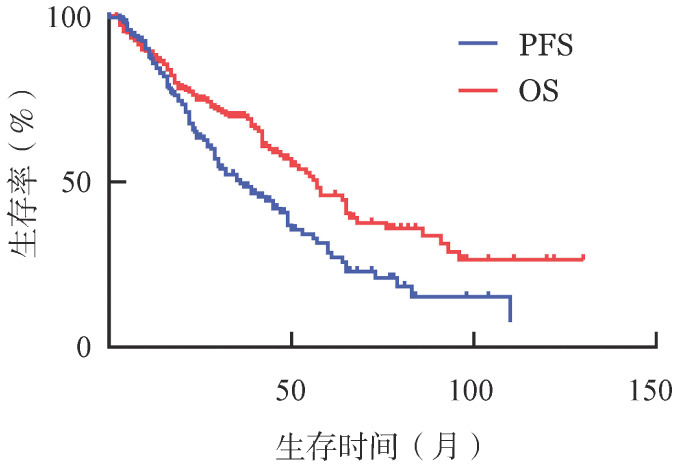
255例老年套细胞淋巴瘤患者的无进展生存（PFS）和总生存（OS）曲线

**表2:** 255例老年套细胞淋巴瘤患者预后的多因素分析

分析指标	无进展生存			总生存	
	*HR*（95%*CI*）	*P*值		*HR*（95%*CI*）	*P*值
年龄≥80岁	1.973（1.110~3.509）	0.021		2.803（1.554~5.056）	0.001
Ann Arbor分期Ⅲ~Ⅳ期	2.806（1.406~5.601）	0.003		1.417（0.677~2.964）	0.355
LDH升高	1.713（1.197~2.451）	0.003		1.892（1.250~2.864）	0.003
骨髓受累	1.617（1.101~2.374）	0.014		1.366（0.878~2.124）	0.166

针对其中50例有白蛋白水平及基础疾病资料的老年MCL患者进行分析，存在基础疾病的患者3年PFS率（17.9％对27.3％，*P*＝0.192）及3年OS率（35.7％对59.1％，*P*＝0.469）低于无基础疾病的患者，但差异无统计学意义。对患者进行预后因素分析，纳入的因素包括是否合并基础疾病、合并基础疾病数目、肾功能、肝功能、糖尿病、高血压、脑血管疾病、乙型肝炎、冠心病、合并其他肿瘤、白蛋白水平。单因素分析显示低白蛋白血症与PFS相关（*P*＝0.028），肝功能不全与PFS（*P*＝0.033）和OS（*P*<0.005）相关。将上述指标及单因素分析中其他*P*值<0.2的指标纳入Cox回归模型进行预后的多因素分析，分析与PFS相关的因素时纳入脑血管疾病（*P*＝0.154）、乙肝（*P*＝0.174）、合并基础疾病（*P*＝0.192），分析与OS相关的因素时纳入乙肝（*P*＝0.198）、合并≥2种基础疾病（*P*＝0.131）；老年患者中年龄、治疗药物的代谢和毒性可能与肾功能相关，故将肾功能同时纳入多因素分析。多因素分析显示，肾功能不全为与OS相关的预后影响因素［风险比率6.206（1.026～37.519），*P*＝0.047］。合并高血压、糖尿病、心脑血管疾病均不是预后的独立影响因素。

## 讨论

MCL是一种好发于老年人的非霍奇金淋巴瘤，具有独特的生物学特点，疾病表现有异质性。既往文献报道，MCL在西方国家中位发病年龄为70岁[2]，以男性居多，常结外受累，骨髓、韦氏环、脾脏及胃肠道受累多有发生，也可累及肾脏、软组织、皮肤及中枢神经系统。在本研究中，老年MCL患者的男女比为3.6∶1，89.4％的患者有骨髓、口咽、脾及胃肠道受累，与以往文献报道相符。本研究中全部患者的中位发病年龄为60岁，老年组患者的中位年龄为69岁，整体发病年龄小于西方国家的文献报道。88.8％的老年患者起病时即为Ⅲ期或Ⅳ期，47.9％的患者基线ECOG评分≥1分，提示老年MCL起病时肿瘤分期晚，一般情况更差，预后不良。

老年患者基础健康状况差，通常有多种合并症，因而该群体对高强度的化疗及自体造血干细胞移植的耐受性更差，在治疗方案的选择中应着重考虑药物不良反应。目前常用于老年MCL的初治方案包括R-CHOP方案、BR方案[3]。在本研究中，大部分（56.9％）患者接受了R-CHOP方案或BR方案，R-CHOP组患者的3年PFS率为23.0％，BR组患者的3年PFS率为31.6％。既往研究表明，包含大剂量阿糖胞苷（≥1 g/m^2^）的方案在小于65岁的年轻MCL患者中疗效良好[4]，但在超过65岁的老年患者中可出现血液系统毒性等严重不良反应[5]。在本研究中，共有43例（16.9％）患者接受了R-CHOP/DHAP、R-HyperCVAD、R-high AraC等包含大剂量阿糖胞苷方案的治疗，3年PFS率和3年OS率分别为30.2％和58.1％。有研究显示包含减量阿糖胞苷（500 mg/m^2^）的方案治疗老年MCL患者的7年PFS率和OS率分别为63％和59％，是老年患者耐受性好且疗效确切的方案[6]。基于上述研究及本研究的结果，在调整剂量和控制不良反应的前提下，包含阿糖胞苷的方案在老年MCL患者中值得临床尝试。以伊布替尼为代表的BTK抑制剂可靶向MCL激活的BCR通路，诱导肿瘤细胞凋亡，增加外周血CD4和CD8阳性T细胞以达到免疫调节的作用。BTK抑制剂伊布替尼与利妥昔单抗联合的IR方案在>65岁的老年MCL患者中的3年PFS率和3年OS率可达到87％和94％，有良好的耐受性和有效性[7]；来那度胺、利妥昔单抗联合的R2方案能使老年和年轻MCL患者获得较高的缓解率和较长的缓解时间[8]；PHILEMON研究则为伊布替尼、来那度胺、利妥昔单抗联合的IR2方案在复发难治的MCL患者中应用提供了支持[9]。在本研究中，将IR、R2与IR2方案作为一组，与R-CHOP或BR方案相比，3年PFS与OS并无显著优势，考虑可能与该治疗组例数少、随访时间短有关。

总体上，老年患者的营养状况差于年轻患者，慢性合并症更多，是一个具有不同活动能力、心理和躯体状态及不同合并症的异质性群体。对于不同基础状态的老年患者，治疗获益及疾病结局有较大的差异。本研究对老年患者的综合疾病评估显示，白蛋白水平、基础肾功能情况与预后相关。对于老年血液系统恶性肿瘤患者，除ECOG评分外，目前有多种评价工具，包括日常生活活动能力（Activities of daily living，ADL）、工具性日常生活活动能力（Instrumental activities of daily living, IADL）、老年风险评估、高龄患者化疗风险评估（Chemotherapy risk assessment for high-age patients，CRASH）。多项研究表明，老年血液系统恶性肿瘤患者的认知受损、高营养风险、合并症多、体能状态差与抗肿瘤治疗不良反应发生率高相关，这部分患者的治疗依从性差，更容易提前终止治疗，其死亡率也更高[10]。应用老年评估来指导临床决策，对于高风险老年人群选用较低强度的治疗方案可提高治疗完成度、减少并发症，获得更好的临床结局[11]。对于老年MCL患者，可在治疗前利用不同的老年评估工具对患者的基线健康状态进行评估，这些评估工具可帮助临床医师针对性地选择治疗方案，以改善MCL患者的整体预后。

综上，老年MCL患者具有基础健康水平差、临床分期晚、预后不佳的特点。在治疗前可通过患者的基线健康水平和临床特点进行预后评估。近年来对于MCL的治疗有了巨大的进展，老年MCL患者有了更多的治疗选择，下一步仍需根据老年MCL患者的基础健康特点探索合适的治疗方案。
